# Development and evaluation of neutralizing antibodies for cross-protection against West Nile virus and Japanese encephalitis virus

**DOI:** 10.1016/j.imj.2023.09.001

**Published:** 2023-09-07

**Authors:** Meng-Jie Yang, Hao-Ran Luo, Zhen-Yu Fan, Yu-Xiang Feng, Ning Wei, Bi-Bo Zhu, Jing Ye, Sheng-Bo Cao, You-Hui Si

**Affiliations:** a National Key Laboratory of Agricultural Microbiology, College of Veterinary Medicine, Huazhong Agricultural University, Wuhan 430070, China; b Hubei Hongshan Laboratory, Wuhan 430070, China; c Frontiers Science Center for Animal Breeding and Sustainable Production, Huazhong Agricultural University, Wuhan 430070, China; d The Cooperative Innovation Center for Sustainable Pig Production, Huazhong Agricultural University, Wuhan 430070, China

**Keywords:** West Nile virus, Neutralizing antibodies, Cross protection, Therapeutic antibodies, Humanization

## Abstract

**Background:**

West Nile virus is a severe zoonotic pathogen that can cause severe central nervous system symptoms in humans and horses, and is fatal for birds, chickens and other poultry. With no specific drugs or vaccines available, antibody-based therapy is a promising treatment. This study aims to develop neutralizing antibodies against West Nile virus and assess their cross-protective potential against Japanese encephalitis virus.

**Methods:**

Monoclonal antibodies against WNV and JEV were isolated by hybridoma technology. The therapeutic efficacy of these antibodies was evaluated using a mouse model, and a humanized version of the monoclonal antibody was generated for potential human application.

**Results:**

In this study, we generated eight monoclonal antibodies that exhibit neutralizing activity against WNV. Their therapeutic effects against WNV were validated both in vivo and in vitro. Among these antibodies, C9-G11-F3 also exhibited cross-protective activity against JEV. We also humanized the antibody to ensure that it could be used for WNV infection treatment in humans.

**Conclusion:**

This study highlights the importance of neutralizing antibodies as a promising approach for protection against West Nile virus infection and suggests their potential utility in the development of therapeutic interventions.

## Highlights

• Eight West Nile virus (WNV) neutralizing antibodies were successfully obtained in this study.
• The antibody C9-G11-F3 demonstrated cross protection against WNV and Japanese encephalitis virus.
• The combination of antibodies B2-D1-H6 and C9-G11-F3 showed a synergistic effect in the treatment of WNV infection in mice.

## Graphical Abstract

**Figure:**
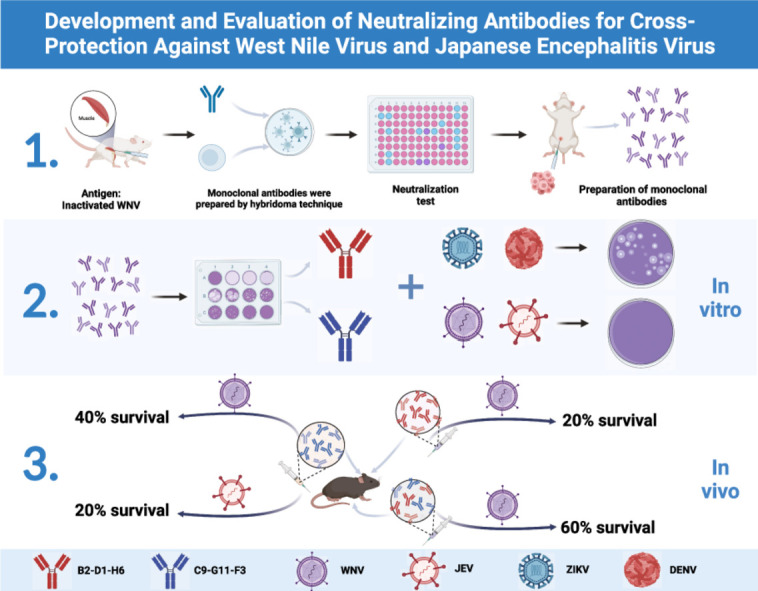

## 1 Introduction

West Nile virus (WNV) is a positive single-stranded RNA virus that belongs to the family *Flaviviridae (Flavivirus)* along with dengue virus (DENV), Zika virus (ZIKV) and Japanese encephalitis virus (JEV) [1]. WNV particles have a spherical shape, with the envelope and capsid showing icosahedral symmetry when observed through cryo-electron microscopy [2], [3], [4]. The WNV genome is composed of a sense single-stranded RNA of approximately 11 kb in length, containing an open reading frame of 109301 nt which encodes 10 proteins, including 3 structural proteins: capsid, membrane and envelop proteins, and 7 nonstructural proteins (NS1, NS2A, NS2B, NS3, NS4A, NS4B, and NS5) [1,^4^,5]. WNV is primarily transmitted through mosquito bites, with *Culex* mosquitoes playing the most significant role in natural transmission. In addition to mosquito-borne transmission, WNV can be transmitted by blood transfusion [6], transplantation [7], breast milk [8] and intrauterine transmission [4,^5^,^7^,^9^,10]. After a susceptible animal is bitten by a WNV carrying mosquito, the virus replicates in dendritic cells before spreading to the local lymph nodes, entering the blood, and invading the nervous system along the peripheral nerve axons. Most people infected with WNV are asymptomatic. However, 20%∼40% of infected people develop West Nile fever, which includes symptoms such as weakness, fatigue, muscle aches, fever, headache, rash, enlarged lymph nodes, vomiting and diarrhea [2]. Among them, about 1% of infected people develop severe neuroinvasive disease, mainly manifested as 3 clinical symptoms of West Nile meningitis, West Nile encephalitis and acute flaccid paralysis. These 3 clinical symptoms can occur simultaneously in the same patient [11]. WNV has been divided into several lineages that do not correspond with geographical distribution. Only lineages 1 and 2 are capable of infecting humans [10].

WNV a significant global public health concern due to its wide distribution across Africa, Europe, Australia, and Asia, as well as its rapid spread throughout the Western hemisphere since 1999, including the United States, Canada, Mexico, the Caribbean, and into parts of Central and South America [12]. The largest reported outbreak of WNV in the United States occurred in August 2012, with 1118 cases reported in 38 states, including 41 deaths. In 2022, 6 additional WNV infection cases were reported in the United States. WNV is a zoonotic infectious pathogen which has caused more than 28,000 reported cases in horses and more than 300 deaths in birds [13]. Crow populations have been reported to decrease by 45% since WNV emerged in the United States [3]. Thus, WNV infection poses a great challenge to global public health.

At present, there are no available specific drugs and commercial vaccines against WNV. While some compounds have demonstrated efficacy in inhibiting WNV at the cellular level, few have displayed antiviral activity in animals [14]. At the same time, due to the limitations of safety and cost, the development of WNV vaccine is also difficult. Therefore, antibody-based therapy is currently considered to be the most promising treatment approach. Studies have shown that neutralizing antibodies against E protein and therapeutic antibodies against NS1 protein can both perform well against WNV at the cellular level and in mice. In particular, the protection provided by WNV neutralizing antibodies plays a key role in WNV treatment [15,16].

In this study, we generated eight monoclonal antibodies that exhibit neutralizing activity against WNV, namely B2-D1-H6, C9-G11-F3, C11-C8-A3, C9-G11-D7, C11-C11-A9, C11-E8-B11, 48-A11-E11 and 48-F12-E10. Their therapeutic effects against WNV were validated both *in vivo and in vitro*. Among these antibodies, C9-G11-F3 also exhibited cross-protective activity against JEV. We also humanized the antibody to ensure that it could be used for WNV infection treatment in humans. This study provides a material basis for the development of WNV therapeutic antibodies, and offers a new antibody cross-reaction strategy for the development of immunotherapeutic reagents for WNVs and other flaviviruses.

## 2 Materials and methods

### 2.1 Viruses and cells

The JEV P3, WNV NY99 and DENV strain was stored in our laboratory. ZIKV H/PF/2013 was obtained from the Microbial Seed Collection Center, Wuhan Institute of Virology, Chinese Academy of Sciences. HEK-293T (human embryo kidney cells) and BHK-21 (baby hamster Syrian kidney cells) cells were maintained in Dulbecco's modified Eagle's medium (DMEM; Gibco) supplement with 10% fetal bovine serum (FBS; Gibco) and incubated at 37 °C in 5% CO_2_. Myeloma cell line SP2/0 were maintained in RPMI-1640 medium (DMEM; BOSTER) supplement with 10% fetal bovine serum (FBS; Gibco) and incubated at 37 °C in 5% CO_2_.

### 2.2 Amplification, inactivation, and ultracentrifugation of WNV NY99

BHK-21 cells were infected with WNV NY99 at 0.1 MOI. When cytopathic effect was observed (36∼48 hours), the supernatant was collected, filtered by 0.22 µm filter membrane, and β-propiolactone at a final concentration of 0.05% was added. The cells were placed at 4 °C for 24 hours and then at 37 °C for 2 hours to hydrolyzate β-propiolactone. The inactivated supernatant was continuously passaged on BHK-21 for 3 generations, and WNV NY99 and β-propiolactone groups were set as controls. The cytopathic effect of each generation was observed under microscope. Subsequently, the inactivated virus solution was centrifuged at 30,000 rpm for 1.5 hours at 4 °C, the supernatant was discarded, and the precipitate was resuspended in 1 mL of sterile DMEM and stored at −80 °C.

### 2.3 Immunization

Immunization of 6-week-old BALB/c female mice was performed according to the immunization procedure presented in Table S1. The 6-week-old BALB/c mice were purchased from the Laboratory Animal Center of Huazhong Agricultural University, Wuhan, China. The serum was collected at day 29, 43, 57, 71, and 85 to determine the neutralization titer. The experimental animal protocols were approved by The Scientific Ethics Committee of Huazhong Agriculture University, ID Number: HZAUMO-2023-0139.

### 2.4 Plaque reduction neutralization test

BHK-21 cells were seeded in 24-well plates at 50,000 cells/well a day early. Serial dilutions of heat-inactivated serum from mice were incubated with 200 plaque forming unit (PFU) of WNV(JEV, DENV, ZIKV) for 90 minutes at 37 °C, then inoculated onto monolayer cells at 37 °C for 60 minutes. Subsequently, the supernatants were removed and incubated with sodium carboxymethyl cellulose (Sigma) containing medium supplemented with 2% FBS. Five days later, the cells were fixed with 10% formaldehyde stained with a crystal violet solution. Plaque numbers were recorded, and a neutralization titer was calculated according to the Reed-Muench method.

### 2.5 Screening of monoclonal antibodies with neutralizing activity

Mouse spleen cells with serum neutralization titer of 1:3200 were fused with myeloma cells SP2/0. The SP2/0 myeloma cell suspension (10^7^ cells) and the immune spleen cell suspension (10^8^ cells) were mixed in a 50 mL centrifuge tube, centrifuged at 1000 rpm for 10 min, discarded the supernatant, and the residual liquid was absorbed by sterilized filter paper. The bottom of the tube was lightly flicked to loosen the cells, and 800 µL of PEG (SIGMA) was slowly added within 1 min in the 37 °C water bath environment, and the edge was gently stirred. Then continue stirring for 1 minute; At 3 minutes, 1 mL of RPMI-1640 basal medium, which was preheated to 37 °C in advance, was added. At 4∼5 minute, 3 mL RPMI-1640 basal medium was added. At 6 minutes, 5 mL RPMI-1640 basal medium was added. Finally, 30 mL RPMI-1640 basal medium was added slowly. After centrifugation at 1000 rpm for 15 minute, the supernatant was discarded and placed at 37 °C for 5∼8 minutes. The cells were resuspended in 40 mL HAT medium and then connected to a previously prepared 96-well plate lined with feeder cells, 100 µL per well, and cultured at 37 °C in a 5% CO_2_ cell incubator.

After successful cell fusion, half of the culture medium was discarded and supplemented with HT medium every other day to observe the size of cell colonies. When 1/4 of the culture wells were reached, the neutralization titer could be determined. Cells with high neutralizing titers were selected for subcloning and repeated 3 times each time. The hybridoma cells that had been subcloned 3 times were established and their neutralizing titers were determined by plaque reduction neutralization assay.

### 2.6 Bulk preparation of monoclonal antibodies

Eight hybridoma cells with high neutralization ability in supernatant were selected for the preparation of monoclonal antibodies. They are B2-D1-H6, C9-G11-F3, C11-C8-A3, C9-G11-D7, C11-C11-A9, C11-E8-B11, 48-A11-E11 and 48-F12-E10. The 10-week-old BALB/c mice were purchased from the Laboratory Animal Center of Huazhong Agricultural University, Wuhan, China. These mice were intraperitoneally injected with 0.5 mL of Freund's incomplete adjuvant, and 3 days later were intraperitoneally injected with 10^4^∼10^5^ hybridomas resuspended in 200 µL RPMI-1640 medium. Microbulging was observed in the abdominal cavity of the mice from the 9th day of injection, and the mice were fed continuously until the abdominal cavity of the mice was round and it was inconvenient to move. The pale yellow liquid was collected into the abdominal cavity of the mice with the needle of a 20 mL syringe. After centrifugation at 1000 rpm for 10 minutes, the supernatant was taken as ascites. The experimental animal protocols were approved by The Scientific Ethics Committee of Huazhong Agriculture University, ID Number: HZAUMO-2023-0139.

### 2.7 Truncated expression and purification of WNV-E protein

WNV E protein was truncated to ED1, ED2, and ED3 for truncated expression. First, according to the nucleotide sequence of WNV E protein that has been published in NCBI, Through B cell epitope prediction software (http://tools.immuneepitope.org/bcell/) and nucleotide affinity-disaffinity water prediction software (https://web.expasy.org/protscale/) the nature of WNV E protein was analyzed. Subsequently, primers were designed according to the nucleotide sequence of WNV E gene published in NCBI (Table S2), and the nucleotide sequence was amplified and connected to the vector pET-30a to construct prokaryotic expression plasmids pET-30a-E, pET-30a-ED1, pET-30a-ED2 and pET-30a-ED3. The selected positive clones were cultured in 10 mL of LB medium at 37 °C and 180 rpm for 12 hours, and then transferred to 1L of sterile LB containing kanamycin at a ratio of 1:100. After the OD_600_ value was between 0.5 and 0.6, 0.5 mM IPTG was added for 5 hours, and the induction was completed. The cells were centrifuged at 7000 rpm for 5 minutes to collect the cells. The collected cells were broken by high pressure at a pressure of 1000 Bar and centrifuged at 12,000 rpm for 20 minutes at 4 °C to collect the inclusion body precipitate. The inclusion bodies were purified by affinity chromatography through a nickel column.

### 2.8 Western blotting

Purified protein samples were taken for western blotting. The samples was run on a 12% Sodium dodecyl sulphate-polyacrylamide gel electrophoresis and transferred to a nitrocellulose membrane. The membrane was incubated with the mAb B2-D1-H6 or C9-G11-F3 and blots were developed using ECL reagents (Thermo Fisher Scientific).

### 2.9 Enzyme-linked immunosorbent assay

We coated 96-well enzyme-linked immunosorbent assay (ELISA) plates (LABSELECT) with 100 µL per well of 10 µg/mL short peptide E2 and subsequently blocked them with 1% BSA in PBS. After washing, we added the antibodies and incubated the plates for 1 hour at room temperature. The bound antibodies were detected using B2-D1-H6. After washing, the plates were incubated for 45 minutes at room temperature using HRP-IgG as a secondary antibody and measured the optical absorbance at 630 nm with an ELISA reader (Thermo Fisher Scientific) according to the manufacturer's instructions.

### 2.10 Animal experiments

The 4-week-old C57BL/6 mice were purchased from the Laboratory Animal Center of Huazhong Agricultural University, Wuhan, China. Mice were randomly assigned into 7 groups. Mice were divided into 5 groups to evaluate the efficacy of WNV treatment: negative serum treatment group, positive serum treatment group, B2-D1-H6 treatment group, C9-G11-F3 treatment group, C9-G11-F3 and B2-D1-H6 combined treatment group. Mice were divided into 2 groups to evaluate the efficacy of JEV treatment: DMEM treatment and C9-G11-F3 treatment group. 10^6^ PFU of challenge WNV, or 10^5^ PFU of JEV were injected intraperitoneally, and B2-D1-H6, C9-G11-F3 were injected intraperitoneally at 20 mg/kg and 5 mg/kg, respectively, for 3 consecutive days after challenge. On the seventh day after challenge, the levels of inflammatory factors and viral load in brain tissues were detected. The experimental animal protocols were approved by The Scientific Ethics Committee of Huazhong Agriculture University, ID Number: HZAUMO-2023-0138.

### 2.11 Humanization of monoclonal antibodies

The variable region genes of monoclonal antibodies B2-D1-H6 and C9-G11-F3 were amplified by PCR. The recovered target bands were linked to the vector pMD 19-T(TAKARA), sequenced, and the structures of antibody variable regions were predicted by antibody structure prediction software (http://www.vbase2.org/). The PCR products of VH and VL genes of monoclonal antibodies B2-D1-H6 and C9-G11-F3 and the heavy chain vector backbone pFUSE2ss-CHIg-hG1 after double digestion with restriction enzymes *Eco*R I and *Bam*H I, respectively, and the antibody after double digestion with restriction enzymes *Eco*R I and *Kpn* I, respectively The backbone of the somatic light chain vector pFUSE2ss-CLIg-hK was ligated by homologous recombination, and the ligated product was transformed into *E. coli* Trans5α to obtain the recombinant plasmid with completely correct nucleotide sequence. The recombinant plasmids of monoclonal antibody C9-G11-F3 were named pFUSE2ss-CHIg-hG1-C9 and pFUSE2ss-CLIg-hK-C9, respectively. The primer sequences utilized in this investigation are presented in Table S2.

## 3 Results

### 3.1 Preparation of inactivated purified WNV and immunization of mice

**Fig. 1:**
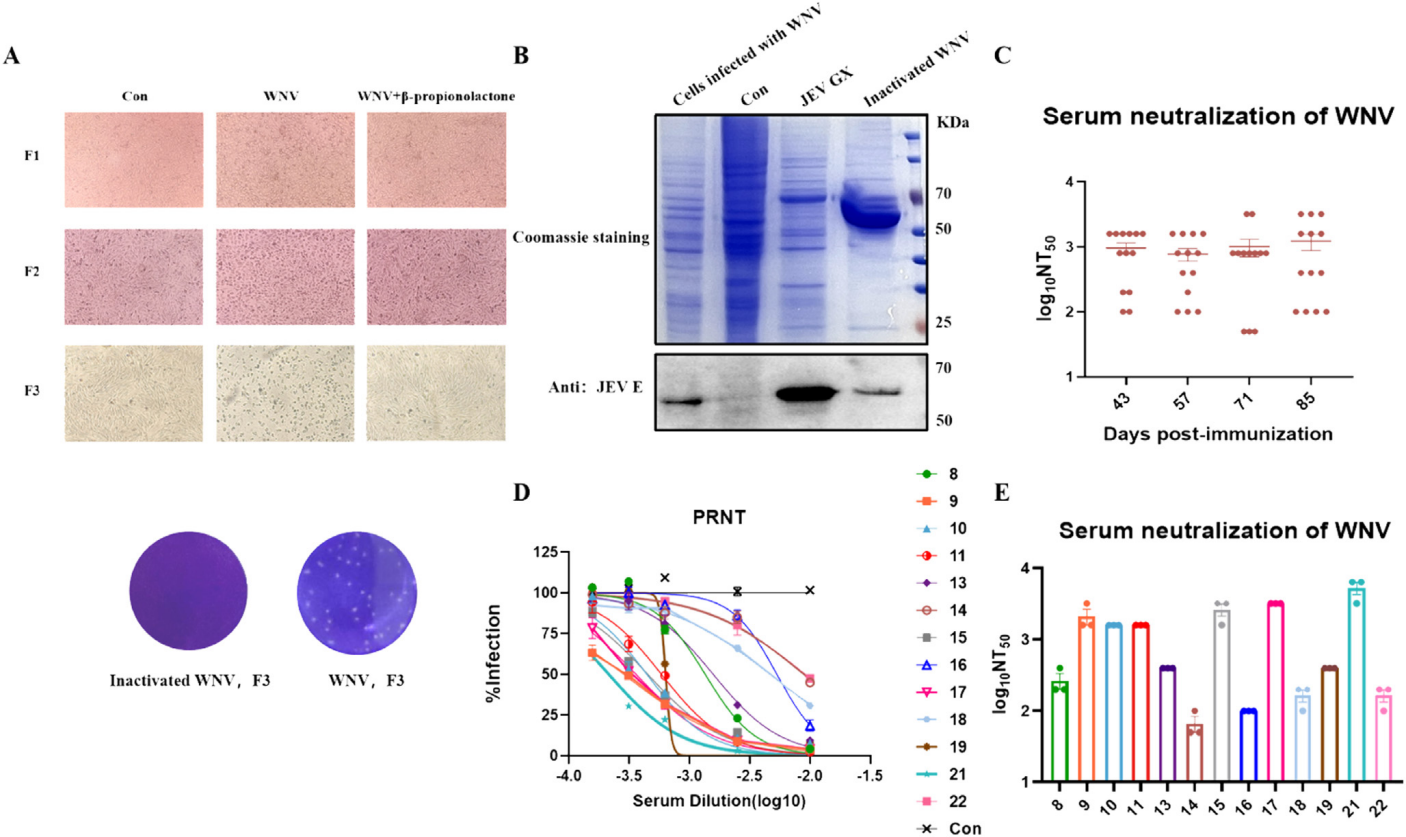
Immunized mouse serum can neutralize WNV. (A) WNV was inactivated using β-propiolactone. The inactivated virus was passaged on BHK-21 cells for 3 consecutive times to ensure no cytopathic effect, demonstrating successful inactivation of WNV. (B) The WNV E protein was stained with Coomassie brilliant blue (top) and analyzed by Western blot (bottom) using monoclonal antibody 1H10 against the E protein of JEV (The E protein of WNV and the E protein of JEV have more than 60% identity, and in a previous study we verified that the antibody 1H10 against the E protein of JEV can also recognize the WNV E protein). The BHK-21 cells infected with WNV at 0.1 MOI for 48 hours and JEV GX virus samples were used as positive controls. Cell specimens without virus infection were used as negative controls. (C) Sera were taken from mice on days 43, 57, 71, and 85 after immunization and assayed for serum inhibition at 50% (NT_50_) dilution of virus infection and each point represents an animal. (D) Dose-response neutralization curves of West Nile virus on day 85 postimmunization against sera from 21 immunized mice Infectivity levels were normalized to those observed in the absence of antibody. “8–22” represents the ear tag of the mouse. Each experiment was independently repeated 3 times and PRNT_50_ was calculated by the Reed–Muench method. (E) Mean values of the reciprocal serum dilution required to inhibit infection by 50% (NT_50_) obtained from serum samples from mice at day 85 and 90 postimmunization. Each experiment was independently repeated 3 times and PRNT50 was calculated by the Reed–Muench method and data are represented as mean ± S.E.M. JEV, Japanese encephalitis virus; WNV, West Nile virus.

To generate a high sera neutralizing antibody titers in immunized mice, we inactivated WNV NY99 using β-propiolactone and then purified it by ultracentrifugation. No cytopathic effect was observed in BHK-21 cells after 3 consecutive passages, while WNV could not produce plaques, which could be determined that WNV NY99 was successfully inactivated (Fig. 1A). To ensure that immunization with this antigen can activate the immune system of the body to induce antibody production, the immunogenicity of this antigen was verified by Western-blot, and the results showed that the purified inactivated E protein in WNV remained, which mean the antigen is immunogenic and can be used for immunization of mice (Fig. 1B). Six-week BALB/c mice were immunized by the antigens prepared above, and sera were collected to detect neutralizing antibody titers after each immunization (Represented by PRNT50) (Fig. 1C). At the same time, we compared the serum titers induced by the inactivated WNV and the prokaryotic expressed and purified WNV E protein as antigen. The results showed that the inactivated WNV as antigen could induce a higher level of WNV neutralizing antibodies, so we used the inactivated WNV as antigen for booster immunization (Fig. S1).The results showed that neutralizing antibody titers in sera from mice after 5 immunizations were high and tended to be stable. Interestingly, 4 mice had high titers of sera PRNT50, reaching 1:3200 (Fig. 1D, E).

### 3.2 Generation of neutralizing antibodies against WNV

We used spleen cells from the above immunized mice which had sera neutralizing antibody titer 1:3200, fused with myeloma cell line SP2/0. After that, Determination of neutralizing antibody titer in hybridoma cell secreting supernatant by PRNT. We selected hybridoma cells with high grade and ability in the supernatant for subcloning, repeated 3 times. Eventually, we selected eight hybridomas with high neutralizing capacity among the hybridoma cells with WNV neutralizing capacity in the supernatant of 32 strains for massive preparation of monoclonal antibodies and subtype analysis of the above eight monoclonal antibodies. The heavy chains were all IgG1 subtype and the light chains were all κ subtype of these eight antibodies (Table 1).

**Table 1:** Identification of monoclonal antibody isoforms.

Monoclonal antibody	Heavy chain	Light chain
B2-D1-H6	IgG1	κ
C9-G11-B6	IgG1	κ
C11-C8-A3	IgG1	κ
C9-G11-D7	IgG1	κ
C11-C11-A9	IgG1	κ
C11-E8-B11	IgG1	κ
48-A11-E11	IgG1	κ
48-F12-E10	IgG1	κ

**Fig. 2:**
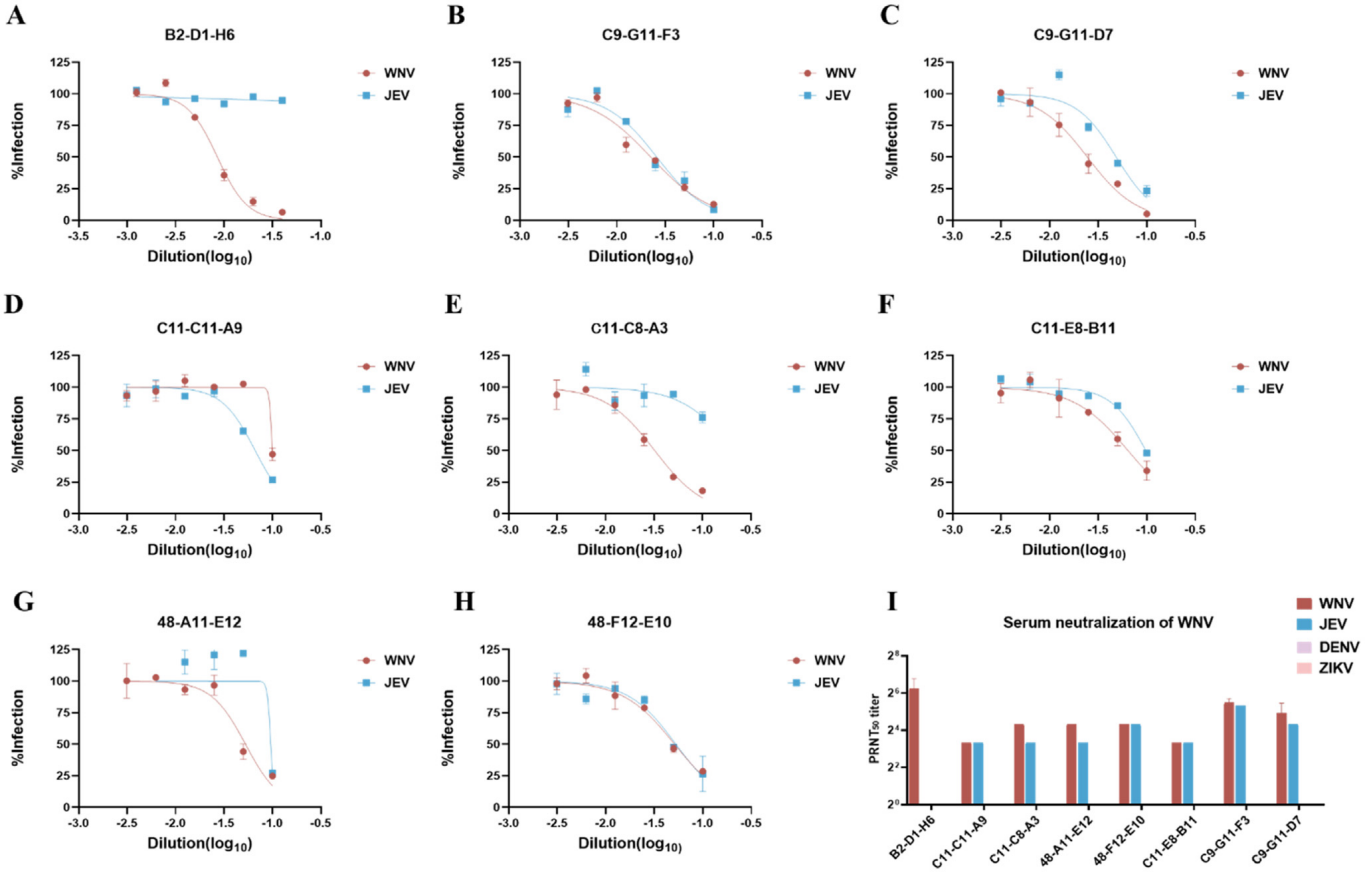
Neutralizing ability of monoclonal antibodies against WNV, JEV, ZIKV and DENV. (A–H) The PRNT method was used to determine the neutralization titers of these 8 mABs against JEV and WNV, and the results were expressed as neutralization curves. (A) B2-D1-H6, (B) C9-G11-F3, (C) C9-G11-D7, (D) C11-C11-A9, (E) C11-C8-A3, (F) C11-E8-B11, (G) 48-A11-E12 and (H) 48-F12-E10. Each experiment was independently repeated 3 times and data are represented as mean ± S.E.M. (I) Mean values of the reciprocal serum dilution required to inhibit infection by 50% (NT_50_) obtained from 8 mABs. Data are represented as mean ± S.E.M. DENV, dengue virus; JEV, Japanese encephalitis virus; mAbs, Monoclonal antibodies; WNV, West Nile virus; ZIKV, Zika virus.

Subsequently, we determined the neutralizing ability of these 8 monoclonal antibodies in vitro by PRNT, as well as the cross-protection ability against other flaviviruses (ZIKV, DENV, and JEV) (Fig. 2A–H). The neutralization curve showed that B2-D1-H6 had only the ability to neutralize WNV(PRNT_50_ = 1:50–1:100), C9-G11-F3, C11-C8-A3, C9-G11-D7, C11-C11-A9, C11-E8-B11, 48-A11-E11 and 48-F12-E10 all had cross-protection capability target JEV, but from PRNT_50_ results, none of these 8 monoclonal antibodies has the ability to cross protect ZIKV and DENV in vitro.

### 3.3 Identification of antibody epitopes

**Fig. 3:**
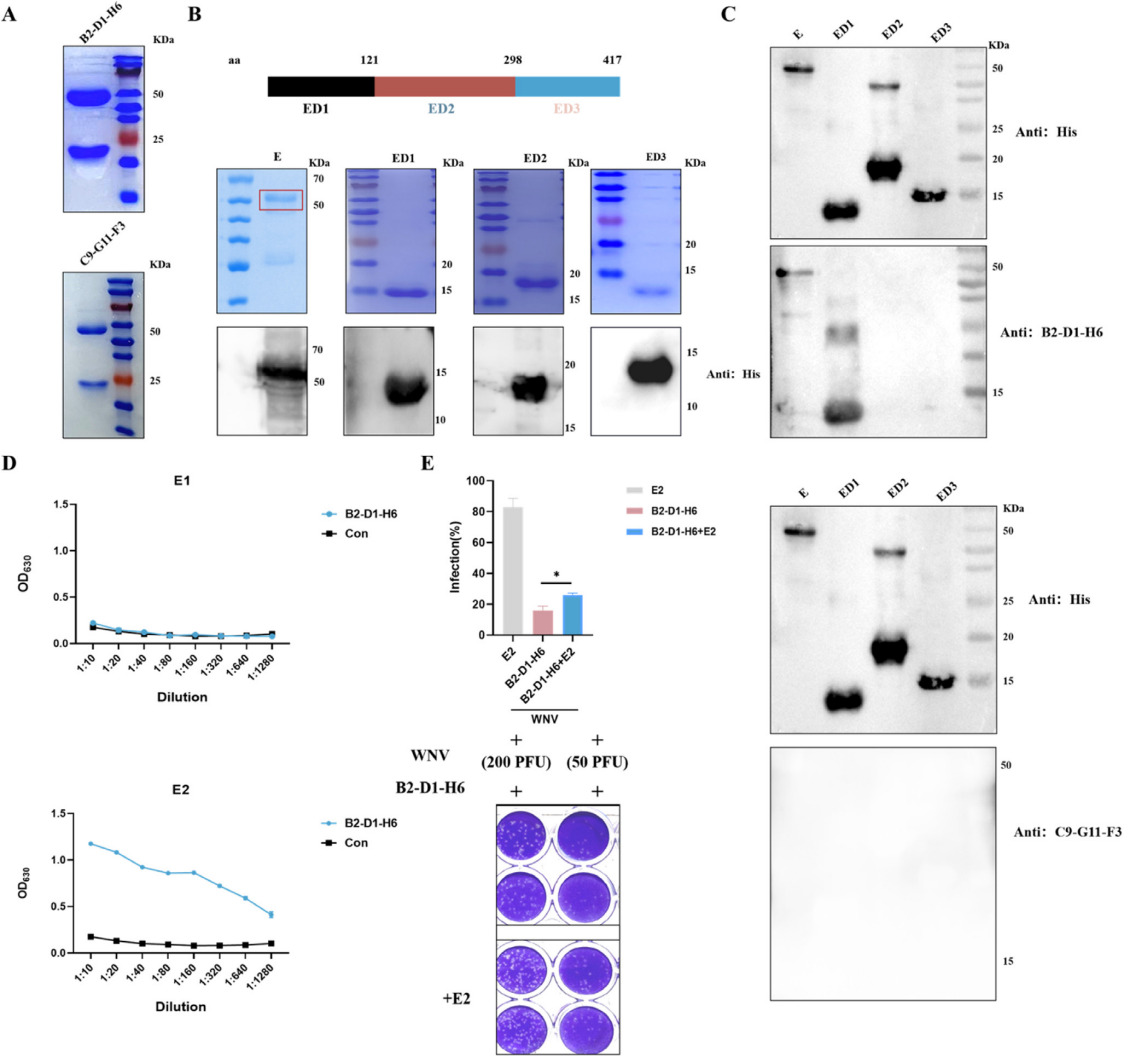
Validation of B2-D1-H6 and C9-G11-F3 targets. (A) Monoclonal antibodies B2-D1-H6 and C9-G11-F3 were purified using Protein A columns and AKTA. Purified antibodies were treated with buffer containing β-mercaptoethanol and analyzed by Coomassie brilliant blue staining on Sodium dodecyl sulphate-polyacrylamide gel electrophoresis. (B) Sodium dodecyl sulphate-polyacrylamide gel electrophoresis and Western-blot analysis of truncated WNV E, ED1, ED2 and ED3 proteins. The antibody used in the Western blotting experiment was His. (C) Truncated expressed proteins was analyzed by Western blotting with the monoclonal antibody B2-D1-H6 and C9-G11-F3. (D) Determination of mAB B2-D1-H6 targeting short peptides E1 and E2 by indirect ELISA. Data are represented as mean ± S.E.M. (*n* = 3). (E) The PRNT method determined that the addition of E2 was able to reduce the neutralization of WNV by mAB B2-D1-H6. The indicated P value was obtained from *t*-test. Data are represented as mean ± S.E.M. (*n* = 3, * *p* < 0.05). WNV, West Nile virus.

We selected B2-D1-H6, which has a high WNV neutralization titer, and C9-G11-F3, which has the best JEV cross protection ability, from the above eight monoclonal antibodies (mABs) to validate their antibody epitopes. To ensure the accuracy of the experiment, monoclonal antibodies B2-D1-H6 and C9-G11-F3 were first purified and quantified using Protein A column (Fig. 3A). Studies have shown that the envelope protein (E protein) was the neutralizing antibody epitope of WNV [17]. To explore which domain of the E protein is affected by the 2 neutralizing antibodies, we truncated them into 3 segments, respectively named ED1, ED2, and ED3 (Fig. 3B). Subsequently, we verified that B2-D1-H6 acts on the first domain ED1 of E protein. While, C9-G11-F3 could not recognize the linear epitope, which might target the spatial epitope (Fig. 3C). To further validate the epitope of B2-D1-H6 and explore which amino acid sequence it acts on the first domain of E protein, we synthesized some short peptides in the first domain ED1 and further validated the epitope of antibody B2-D1-H6 by ELISA. The results showed that the epitope of B2-D1-H6 was a short peptide E2 with a length of 20 amino acids in the first domain of E protein, and the sequence was VCRQGVVDRGWGNGCGLFGK (Fig. 3D). To further verify that short peptide E2 is indeed the epitope of B2-D1-H6, we conducted an E2 blocking test. Firstly, short peptide E2 was interacted with B2-D1-H6 to block the neutralizing epitope on the surface of B2-D1-H6, and then the neutralizing effect of B2-D1-H6 against WNV after E2 blocking was measured using the PRNT method. The results showed that the neutralization titer of B2-D1-H6 against WNV decreased after blocking with short peptide E2 (Fig. 3E). All the above suggested that the epitope of monoclonal antibody B2-D1-H6 was a short peptide with a length of 20 amino acids in the first domain of E protein, while monoclonal antibody C9-G11-F3 could not recognize linear epitopes, which might recognize spatial structures.

### 3.4 Therapeutic effect of mABs against WNV

**Fig. 4:**
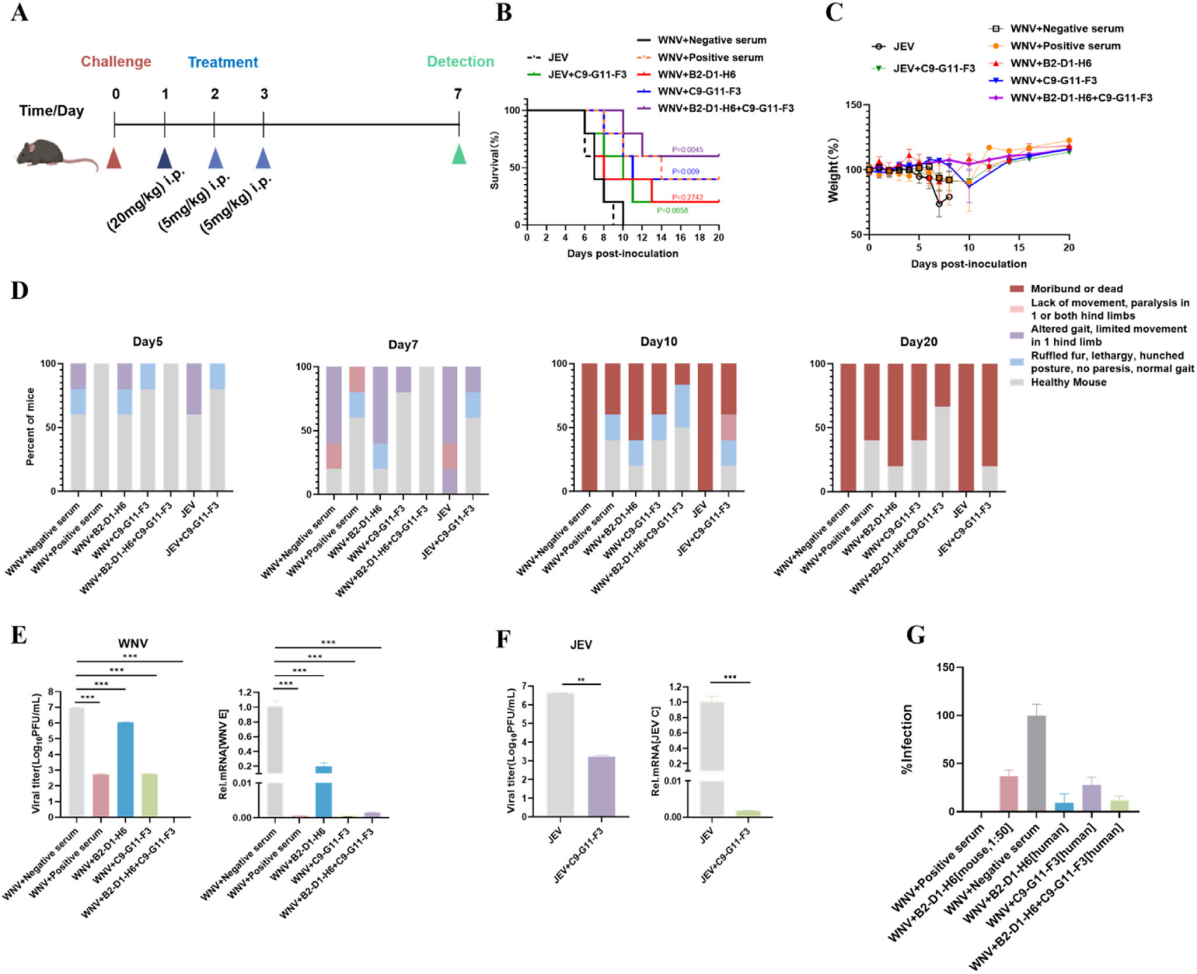
Therapeutic efficacy of mAbs. (A) Four-week-old C57/BL6N mice were inoculated with 10^6^ PFUs of WNV, and mAB B2-D1-H6, C9-G11-F3 or both mABs were injected by intraperitoneal injection of 20 mg/kg, 5 mg/kg, and 5 mg/kg on days 1, 2, 3 after challenge. A negative serum treatment group and a positive serum treatment group were also set up as controls, and each group contained 10 mice. (B) Survival rate. (C) Body weight changes and (D) behavioral score were recorded after WNV NY99 strain challenge. Survival rate is displayed as Kaplan–Meier survival curves and behavioral changes from normal to severe are represented on a scale of 0 to 4. (E,F) Viral loads in mice brain were detected by plaque assay and qRT-PCR at day 7 after infection. (E) WNV, (F) JEV. Data are analyzed using an ANOVA and represented as mean ± S.E.M (no significances *p* > 0.05, *p < 0.05, **p < 0.01, ***p < 0.001). (G) Humanization of murine-derived mABs and determination of their PRNT_50_. The humanized antibody was concentrated 5-fold by ultracentrifugation, and the murine antibody was used as a positive control. JEV, Japanese encephalitis virus; mAbs, Monoclonal antibodies; PFU, Plaque forming unit; WNV, West Nile virus.

To evaluate the effectiveness of the 2 neutralizing antibodies mentioned above in treating WNV infection in mice, we injected the monoclonal antibodies prepared continuously on the first, second, and third days after the treatment. On the seventh day, we took mouse brain tissue for viral load analysis (a negative sera treatment group as a negative control, and a positive sera treatment group as a positive control) (Fig. 4A). The body weight (Fig. 4C), clinical behavior score (Fig. 4D), and protection rate (Fig. 4B) of the mice were statistically analyzed. The results showed that the negative control group mice began to lose weight on the 4th day and continued to lose weight on the 10th day until all the mice died. In addition, the mice in the antibody C9-G11-F3 and B2-D1-H6 treatment groups experienced a slight decrease in body weight from the 4th day, and gradually regained weight after the 6th day. At the same time, the mice in the antibody treatment group had lower clinical symptom scores. Compared to the negative sera treatment group, monoclonal antibody B2-D1-H6 has a 20% protection rate against WNV infection in mice, and monoclonal antibody C9-G11-F3 has a 40% protection rate against WNV infection in mice. It is worth noting that the combined use of these 2 antibodies can achieve a protective effect of 66.7%, indicating that these 2 antibodies have a synergistic effect. The measurement results of viral load in mouse brain tissue also showed that both B2-D1-H6 and C9-G11-F3 as well as the combined treatment group could significantly reduce viral load in mouse brain tissue (Fig. 4E).

Mouse derived monoclonal antibodies can cause rejection reactions in humans, thereby affecting the therapeutic effectiveness of antibodies. Therefore, we amplified the sequences of the heavy chain variable region (VH) and light chain variable region (VL) of the above 2 antibodies and connected them to the modified humanized antibody skeleton plasmids pFUSEss-CHIg-hG1 and pFUSEss-CLIg-hK. We constructed a humanized recombinant antibody plasmid, transfected it into HEK-293T cells, and collected the supernatant to verify the neutralizing effect of the recombinant antibody. The results show that humanized B2-D1-H6 and C9-G11-F3 still have neutralizing ability against WNV (Fig. 4G).

### 3.5 Cross-reactivity to JEV

In previous experiments, we verified that the monoclonal antibody C9-G11-F3 has the cross protection ability against JEVs *in vitro*. To further verify whether it has the protection ability against JEVs *in vivo*, we injected C9-G11-F3 for 3 consecutive days for treatment, and counted the weight (Fig. 4C), clinical behavior score (Fig. 4D) and protection rate (Fig. 4B) of the mice from 0–20 day. The results showed that the challenge JEV group mice began to lose weight on the 4th day and continued to lose weight on the 9th day until all the mice died. The weight of mice in the C9-G11-F3 treatment group decreased slightly from day 4, and gradually increased after day6. The C9-G11-F3 treatment group mice had lower clinical symptom scores, and the monoclonal antibody C9-G11-F3 had a 20% protection rate against JEV infection in mice.

### 3.6 mABs treatment attenuates the neuroinflammatory response induced by WNV infection in mice

**Fig. 5:**
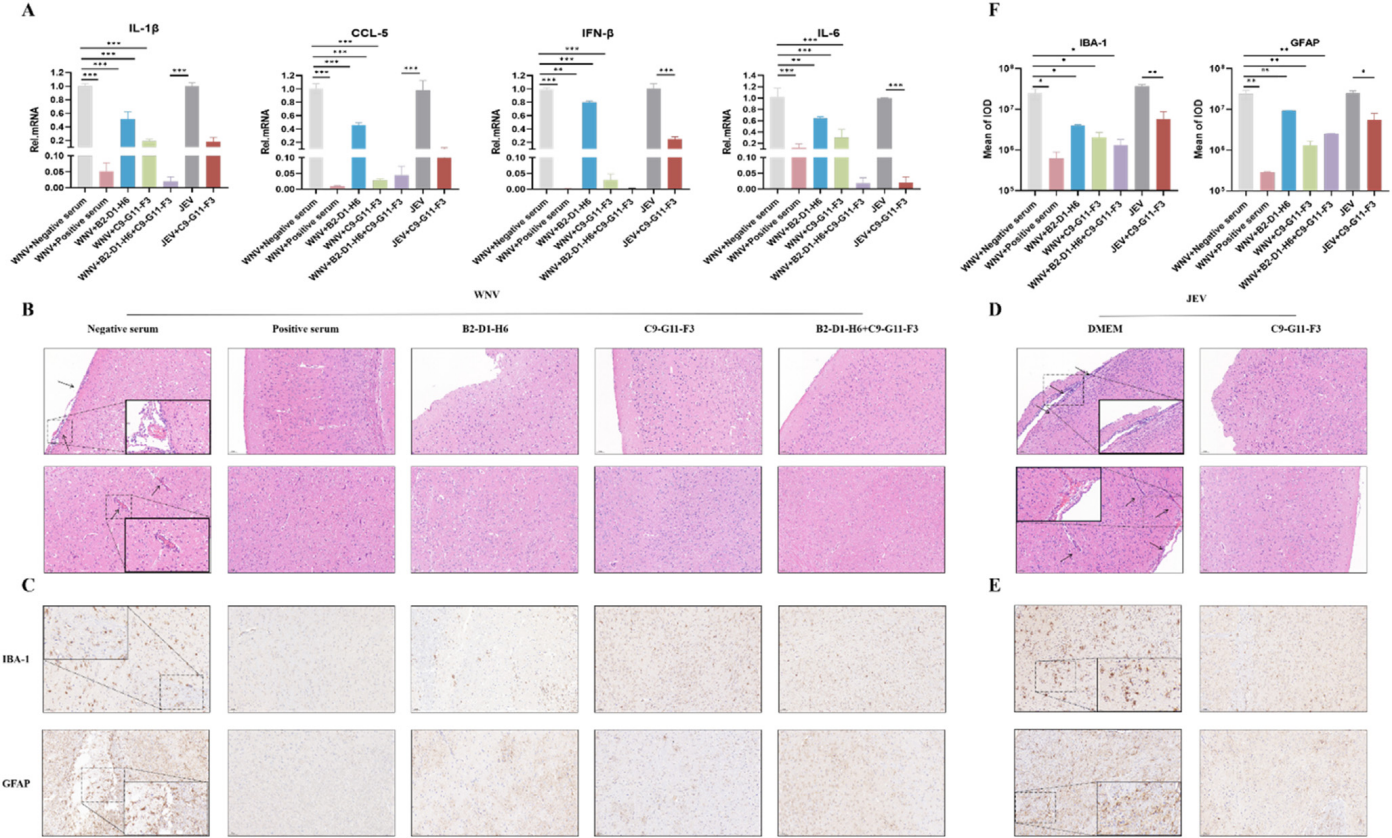
mABs treat WNV or JEV induced inflammatory responses in the mouse brain. (A) The mRNA expression levels of inflammatory cytokines (IL-6, IL-1β, CCL-5, TNF-α) in brain tissue lysates were quantified by qRT-PCR. Data are analyzed using an ANOVA and represented as mean ± S.E.M (*n* = 3, no significances *p* > 0.05, *p < 0.05 , **p < 0.01, ***p < 0.001). (B–E) The activation of the pathological changes and glial cells in brain tissue of mice were analyzed by H&E staining and Immunohistochemistry. The arrows in (B) indicate areas with severe inflammatory cell infiltration and vascular cuffing. (F) Integrated option density analysis of 3 visual fields of slides from each group was performed to quantify the results of immunohistochemical staining (the lower panels). Scale bar = 50 µm. Data are represented as mean ± S.E.M. (*n* = 3; ns, no significance; *p* > 0.05, *p < 0.05 , **p < 0.01, ***p < 0.001). JEV, Japanese encephalitis virus; mAbs, Monoclonal antibodies; WNV, West Nile virus.

Given the ability of B2-D1-H6 and C9-G11-F3 to protect mice from lethal WNV challenge, we next evaluated whether the prepared monoclonal antibodies could prevent the associated inflammatory response in the mouse brain. On day 7 after the WNV challenge, the brain tissues of 3 mice from each group were collected. The expression of inflammatory cytokines, including IL-1β, IL-6, CCL5 and IFN-β were measured by RT-qPCR. The results showed that WNV infection caused a massive expression of inflammatory cytokines, and mABs could significantly reduce the expression of inflammatory cytokines (Fig. 5A). In addition, the WNV caused the pathologic changes and gliosis were examined by HE and Immunohistochemistry staining, respectively. As shown in the HE staining results, in the brains of mice inoculated with negative sera, blood vessels were highly congested, and a large number of infiltrated inflammatory cells and vascular sleeves were observed (Fig. 5B). In addition, nodules of glial cells, neuronal vacuolar necrosis and nuclear metachromasia were also shown in this group. However, no obvious pathological changes were observed in the brains of mice in the mABs treatment groups. The Immunohistochemistry results revealed that microglia and astrocytes in negative sera treatment group showed an activated state of antennae-like expansion and a darker brown cytoplasm. It also indicated that C9-G11-F3 treatment could reduce the neuroinflammatory response induced by JEV infection in mice (Fig. 4F, Fig. 5D–F). Based on the above results, C9-G11-F3 has the ability to cross protect JEVs. In contrast, mice treated with mABs showed no obvious changes in the morphology of glial cells. The integrated option density analysis of the images also confirmed the observation (Fig. 5C, F). Taken together, these results suggest that the neutralizing antibodies we prepared could largely attenuate a WNV-induced inflammatory response in mice brains.

## 4 Discussion

Antibodies have been used as therapeutic drugs for a long time, particularly in the prevention and treatment of COVID-19. In the case of SARS-CoV-2, neutralizing antibodies targeting the S protein have played a crucial role in its treatment [18], [19], [20]. Studies have demonstrated that neutralizing antibodies against E protein and therapeutic antibodies against NS1 protein exhibit positive effects in the treatment of WNV at both the cellular level and in mice [14], [15], [16],21]. Specifically, neutralizing antibodies targeting the E protein have shown remarkable protection in animals and have demonstrated efficacy in the treatment of human WNV infection. The efficacy of antibody therapy is primarily influenced by the neutralizing titer and affinity of antibodies [22]. WNV envelope protein plays crucial roles in various physiological functions, including cell communication and the induction of neutralizing antibodies [23]. In this study, maintaining the spatial structural stability of WNV envelope proteins was essential to elicit higher levels of neutralizing antibody titers. Ideally, a eukaryotic expression system is preferred for expressing and purifying the envelope protein in large amounts for mouse immunization and subsequent verification of monoclonal antibody epitopes. However, due to the unique characteristics of WNV envelope E protein structure, we failed to obtain sufficient yield using the eukaryotic expression system. As a result, we resorted to using a prokaryotic expression system to express WNV envelope proteins [24,25]. We compared serum neutralization titers generated in mice immunized with spatially structured WNV inactivated virus solution vs those immunized with prokaryotic expressed WNV E protein. The results showed that neutralization titers induced by the use of inactivated WNV as antigen were more than 10-fold higher than those induced by the serum of mice immunized with prokaryotic expressed WNV E protein (Fig. S1). This result further emphasizes that compared with the prokaryotic expression system, the eukaryotic expression system is more advantageous in maintaining the accurate spatial conformation and facilitating the induction of neutralizing antibodies [26], [27], [28], [29], [30].

In this study, we screened 32 hybridoma cell strains to identify those producing supernatant with high WNV neutralizing titers, and 8 strains with high neutralizing titers were selected for mass production of monoclonal antibodies. We further investigated the relationship between the epitopes, neutralizing titers and antiviral effects in animals of 2 antibodies B2-D1-H6 and C9-G11-F3. Western blot and ELISA results confirmed that the B2-D1-H6 epitope is a 20 amino acid short peptide in the first domain of E protein, with the amino acid sequence VCRQGVVDRGWGNGCGLFGK. C9-G11-F3 does not recognize linear epitopes but potentially targets spatial epitopes [17]. Previous studies have shown that monoclonal antibodies against the lateral ridge of ED3 protein offer effective protection against WNV infection in animals. By utilizing phage display of WNV E protein variants, researchers have identified 25 E protein residues that are critical for recognition by ED1 or ED2-specific neutralizing monoclonal antibodies. These neutralizing epitopes do not confer cross-protect against other flaviviruses, such as DENV. In other words, monoclonal antibodies against the WNV ED1 and ED2 can protect mice from WNV infection and do not have the ability to cross-protect other flaviviruses [31]. Our study's findings align with these results, as B2-D1-H6 monoclonal antibody targeting ED1 does not provide cross-protection against other flaviviruses. In mice, C9-G11-F3 had a good protective effect against WNV infected mice with a protection rate of 60%. Additionally, this antibody exhibited cross-protection effect against JEV infection in mice with a cross-protection rate of 20%. The protection rate of B2-D1-H6 against WNV infection was 20%. C9-G11-F3 showed better therapeutic effect in mice *in vivo*, potentially due to its recognition of spatial epitopes. Previous reports have indicated that WNV-86, a neutralizing antibody of WVV with high titer, preferentially recognizes virions with spatial epitopes *in vivo* [22]. These findings further support the importance of spatial structure for both neutralizing antibody titer and antibody therapeutic efficacy. Our results align with previous observations that employing an antigen with spatial structural activity can induce higher levels of neutralizing antibody titers in mouse serum. Moreover, our study found that the combination of 2 specific monoclonal antibodies has synergistic effect for the treatment of WNV. This suggests that utilizing a cocktail therapy that combines different antibodies holds even greater potential and effectiveness.

It has been reported that some compounds (multiple cyanohydrazones, etc.) can block the entry of DENV, ZIKV, and JEV by inhibiting flavivirus E protein-mediated fusion. However, most of these compounds demonstrate viral inhibition at the cellular level, with limited antiviral activity in animals [14,^32^,33]. Meanwhile, monoclonal antibody 1G5.3 directed against WNV NS1 protein, has been effective in blocking multiple flavivirus NS1-mediated cell permeability. Therapeutic application of 1G5.3 reduced viremia and improved survival in murine models of dengue, Zika, and WNV [15]. Combining these findings with the results of the current study, it suggests that developing broad-spectrum neutralizing antibodies against flaviviruses holds high feasibility and application potential.

Although there have been no cases of WNV infection in China so far, it has spread to North America and other regions, causing infections worldwide. This has brought a huge impact on the local medical system and public health. Unfortunately, there are currently no specific drugs and commercial vaccines available for WNV. While some compounds have shown inhibitory effects on WNV at the cellular level, their effectiveness in animals is limited [21]. Due to the limitations of safety and cost, the research and development of WNV treatments also present challenge. Consequently, antibody therapy is currently the most potential treatment for WNV infection [34,35]. However, the murine monoclonal antibody prepared in this study may elicit an immune response when administered to human with WNV infection. Therefore, it is necessary to humanize the monoclonal antibody enhance their efficiency and safety. The use of single B cell technology for the production of fully humanized antibodies proves to be more advantageous. However, for infectious diseases such as West Nile encephalitis that have not emerged in China but pose high potential risks, directly obtaining biological samples from infected patients for the production of therapeutic antibodies using single B cell technology is challenging. In this study, the VH and VL sequences of monoclonal antibodies C9-G11-F3 and B2-D1-H6 were amplified and cloned into modified humanized antibody expression backbone vectors pFUSE2ss-CLIg-hG1 and pFUSE2ss-CLIg-hK, respectively. These constructs were then transfected into HEK-293T cells to produce humanized recombinant antibodies. The cellular level verification confirmed that the recombinant humanized antibody retained their neutralizing ability against WNV [35], [36], [37], [38], [39]. Subsequently, we aim to further validate the therapeutic efficacy of the recombinant humanized antibody against WNV in a mouse model. However, given the suboptimal neutralizing ability of the recombinant antibody and concerns regarding animal welfare policy, we refrained from verifying the therapeutic effect of the humanized antibody in mice. Moreover, it is of paramount importance to conduct further studies to determine whether the harvested recombinant antibodies can effectively treat human WNV infection.

In summary, our study successfully screened and produced neutralizing antibodies against WNV and cross-protective neutralizing antibodies against JEV, and validated their therapeutic effects both *in vitro* and *in vivo*. Additionally, we humanized the murine monoclonal antibodies generated in the laboratory to ensure their therapeutic efficacy for treating WNV and JEV infection in humans. Our study not only provides a material basis for the prevention and treatment of WNV and JEV, but also highlights potential utility of neutralizing antibodies as a promising approach in the development of strategies for the prevention and control of imported diseases in China.

## Appendix Supplementary materials

Image, application 1
