# Supplementary material for: Development and evaluation of neutralizing antibodies for cross-protection against West Nile virus and Japanese encephalitis virus
Source: Infect Med (Beijing). 2023 Sep 7;2(3):212–23. doi: 10.1016/j.imj.2023.09.001 (PMC10699678; doi:10.1016/j.imj.2023.09.001)
Supplement: Supplementary file 1 [file mmc1.docx]

**Fig. S
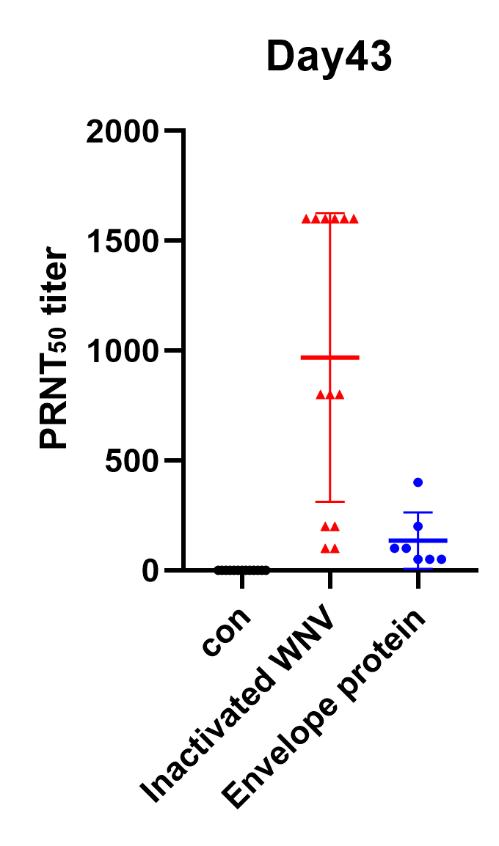
1**

**Table. S1** Immunization programs in mice

| Time (d) | Pathway | Dosage (μg) | Adjuvant |
| --- | --- | --- | --- |
| 1 | Intramuscular injection | 200 | Freund's complete adjuvant |
| 15 | Intramuscular injection | 200 | Freund's incomplete adjuvant |
| 29 | Intramuscular injection | 200 | Freund's incomplete adjuvant |
| 43 | Intramuscular injection | 200 | Freund's incomplete adjuvant |
| 57 | Intramuscular injection | 200 | Freund's incomplete adjuvant |

**Table. S2**  Primers used in this study.

| Genes | Primer sequences (5’-3’) |
| --- | --- |
| WNV E | F:CGCGGATCCATGTTCAACTGCCTTGGA |
|  | R:CCCTCGAGGATGGTGATGGTGATGATGTCTCTGCGCTCCTTT |
| WNV ED1 | F:CGCCATATGTTCAACTGCCTTGGAATGAGC |
|  | R:CCGCTCGAGGCAGGCAAATTTGGCGCATGT |
| WNV ED2 | F:CGCCATATGTGCTCTACCAAGGCAATAGGA |
|  | R:CCGCTCGAGCTTCAACTGCAATTTTTCCAT |
| WNV ED3 | F:CGCGGATCCATGAAGGGAACAACCTATGG |
|  | R:CCCTCGAGGATGGTGATGGTGATGATGCGCTCCTTTGAGGGTGGT |
| mIL-6 | F:AACGATGATGCACTTGCAGA |
|  | R:GAGCATTGGAAATTGGGGTA |
| mIL-1β | F:TGTCTGAAGCAGCTATGGCAAC |
|  | R:CTGCCTGAAGCTCTTGTTGATG |
| mCCL5 | F:GCCCACGTCAAGGAGTATTTCTA |
|  | R:ACACACTTGGCGGTTCCTTC |
| IFN-β | F:GTCCTCAACTGCTCTCCACT |
|  | R:CCTGCAACCACCACTCATTC |
| mβ-actin | F:CACTGCCGCATCCTCTTCCTCCC |
|  | R:CAATAGTGATGACCTGGCCGT |
| JEV P3 C | F:TGGCAGAAGCTTTGAGAGGG |
|  | R:TATACTGGCTGGGTCGGTGA |
| WNV NY99 E | F:TTCTCGAAGGCGACAGCTG |
|  | R:CCGCCTCCATATTCATCATC |
